# Therapeutically Attenuating Neutrophil Recruitment With a CXCR2 Antagonist in Combination With Oseltamivir Ameliorates Influenza-Induced Lung Injury and Disease

**DOI:** 10.1093/ofid/ofz106

**Published:** 2019-03-07

**Authors:** Michael L Washburn, Renae Crosby, Katja Remlinger, Feng Wang, Donald Creech

**Affiliations:** 1 GlaxoSmithKline, Upper Providence, Pennsylvania; 2 GlaxoSmithKline, Research Triangle Park, North Carolina

**Keywords:** biomarker, CXCR2 antagonist, influenza, neutrophils

## Abstract

Mice were infected with influenza and treated with a CXCR2 antagonist in combination with antiviral or antiviral alone starting 4 days postinfection. Neutrophil recruitment to the lung was reduced, and improvements in health outcomes and lung consolidation were observed in combination-treated mice with no evidence of worsening outcome.

Influenza is a significant global health threat that causes substantial morbidity and mortality. Despite the availability of vaccines and direct-acting antiviral (DAA) drugs, influenza continues to cause severe disease and remains a significant burden on the global healthcare system [1]. Although DAA agents are able to reduce viral load, they need to be given early in the course of disease to have an effect and have not prevented death in patients with severe influenza [2].

Pathogenesis of influenza results not only from direct viral cytopathology but also from an overzealous host immune response, leading to lung inflammation and decreases in lung function. Neutrophils are identified as a key cell type leading to deteriorating lung function through the production of reactive oxygen species, mucus hypersecretion, neutrophil extracellular traps, and tissue-destructive proteinases [3]. Respiratory neutrophil levels and their associated chemokines involved in recruitment to sites of inflammation are correlated with clinical symptom severity of influenza infection in humans, in particular in those who died [4, 5]. The data suggest that at the time of hospitalization, excessive host-driven inflammation is a major contributor to disease pathology and worsening outcomes [6]. Recent development of animal models of influenza infection that recapitulate human disease highlight the detrimental impact of excessive neutrophil recruitment to the lung, and together these studies suggest that attenuating neutrophil recruitment to the lungs may be an effective therapy to reduce morbidity and mortality from severe influenza infections [7, 8].

 Neutrophils are activated and recruited towards sites of inflammation through chemokine signaling of the CXCR2 pathway [9]. SB-332235Z is a potent and selective CXCR2 antagonist that prevents binding of CXC ligands, such as in interleukin (IL)-8, and, in preclinical studies, it is able to reduce neutrophil chemotaxis to the lungs and sites of inflammation [10]. In this study, we explored the ability of the CXCR2 antagonist SB-332235Z to reduce excessive neutrophil accumulation and subsequent inflammatory damage in the lungs of influenza-infected mice.

## METHODS

Female BALB/cAnNCrl mice were infected intranasally with influenza (A/PR/8/34; 25 TCID_50_). Starting on Day 4 postinfection, mice were dosed twice daily by oral gavage with vehicle (placebo), 10 mg/kg oseltamivir phosphate (OSV), or a combination of both OSV and 40 mg/kg CXCR2 antagonist SB-332235Z. All studies were conducted in accordance with the GSK Policy on the Care, Welfare and Treatment of Laboratory Animals and were reviewed by the Institutional Animal Care and Use Committee either at GSK or by the ethical review process at the institution where the work was performed.

Throughout the course of the experiments, mice were monitored daily for clinical scores (0–3 scale), body weight, and blood oxygen saturation. Groups of mice were euthanized on Day 7, 10, or 14 to measure various endpoints. On Day 7 postinfection, bronchoalveolar lavage fluid (BALF) was collected for cellular fluorescence-activated cell sorting (FACS) analysis and chemokine measurement. Lung tissue was collected for viral load quantitation by quantitative polymerase chain reaction (qPCR). The lobes of the lungs were separated, dissected, and weighed before processing. One third of the left lobe was placed in a ribonucleic acid (RNA) stabilization solution in a 2-mL eppendorf tube precooled to 4^o^C for subsequent RNA extraction and influenza virus (IFV) RNA quantitation. The tissue sample was held at 4^o^C for 24 hours and then stored at −80^o^C. The IFV-positive strand complementary deoxyribonucleic acids (cDNAs) were generated using the SuperScript^TM^ III reverse transcriptase and strand-specific reverse-transcription primers containing tag sequences. In the subsequent qPCR reactions, the tags were recognized by a tag-specific qPCR primer, reducing the detection of nonspecific cDNAs. The positive strand standard RNAs were generated by in vitro transcription using the Riboprobe^TM^ System-T3 according to the manufacturer’s directions. The levels of IFV A/PR/8/34 RNA in the lung tissue samples were quantitated by strand-specific qPCR (ssqPCR): positive strand reverse-transcription primer 5’-CGGTCATGGTGG CGAATAACTCATCGCT TGCACCATTTG-3’; positive ssqPCR primer 5’-CAGCACTAC AGCTAAGGCTATG-3’; ssqPCR Tag primer 5’-CGGTCATGGTGGCGAATAA-3’; ssqPCR probe 5’-(56-FAM)CCTCTGCTG(Zen) CTTGCTCACTCGATC(3IABkFQ)-3’.

The right lobe was placed in 1 mL dissociation buffer (Dulbecco’s modified Eagle’s medium supplemented with 10% fetal bovine serum [FBS], nonessential amino acids, l-glutamine, penicillin/streptomycin, 150 U/mL collagenase type IV, and 2 U/mL DNaseI) in gentleMACS C tubes and held on ice before processing for subsequent FACS analysis.

Neutrophils from BALF were analyzed by FACS. Bronchoalveolar lavage fluid was filtered through a MultiScreen-Mesh filter plate, and red blood cells were lysed with ammonium-chloride-potassium lysis buffer. The cells were washed once with phosphate-buffered saline containing 2% FBS and resuspended in an antibody mixture (antimouse Ly-6G, CD11b, CD45 [BioLegend], and LD7 [Life Technologies] antibodies). The percentage of neutrophils (Ly-6G-positive, CD11b-positive) of the live (LD7-negative) CD45-positive leukocytes was determined on a Gallios FACS machine (Beckman Coulter) and analyzed using FlowJo software (version 10; Tree Star, Inc.). A custom Milliplex MAP Mouse Cytokine/Chemokine Magnetic Bead Panel was used to quantitate the level of KC and RANTES in the BALF. The assay plates were read on a MAGPIX multiplexing instrument (Millipore) using xPONENT software, and the amount of each analyte present was determined from a standard curve. On Day 14, a group of mice was euthanized for lung histopathological assessment to quantify the extent of alveolar consolidation in the lung parenchyma.

For statistical analysis, the data were analyzed in SAS 9.2 (SAS Institute, Cary, NC) and graphed in GraphPad Prism 7.02 with error bars representing standard error of the mean. The area under the curve (AUC) change from baseline weight was calculated as follows: for each animal, body weight was subtracted from the Day 0 baseline weight, and the AUC was then calculated across Days 0 to 10. Likewise, AUC change from baseline oxygen saturation was calculated by first subtracting the oxygen saturation from the Day −2 baseline oxygen saturation and then calculating the AUC across Day −2 to Day 9. Day −2 was used as the baseline measure, because oxygen saturation was not measured on Day 0. The average clinical score was calculated by taking the average across clinical scores from Day 3 to Day 9 for each animal. To fulfill analysis assumptions, a log_10_ transformation was applied to KC, RANTES, and viral load measures. Using PROC MIXED [11], a linear model with a fixed effect for treatment (and different residual variances for each level of treatment for some endpoints) was fit, followed by all pair-wise comparisons. Comparing OSV with OSV^+^, ‘235 was of primary interest, and therefore no multiplicity adjustment was applied, and unadjusted *P* values were reported. For analysis of the lung alveolar consolidation, in addition to the analysis described above, a 2-sample *t* test was performed to compare the OSV monotherapy to combination therapy, and no multiplicity adjustment was applied. The data are representative of 2 independent experiments with 5–6 animals per group.

## RESULTS

**Figure 1.**
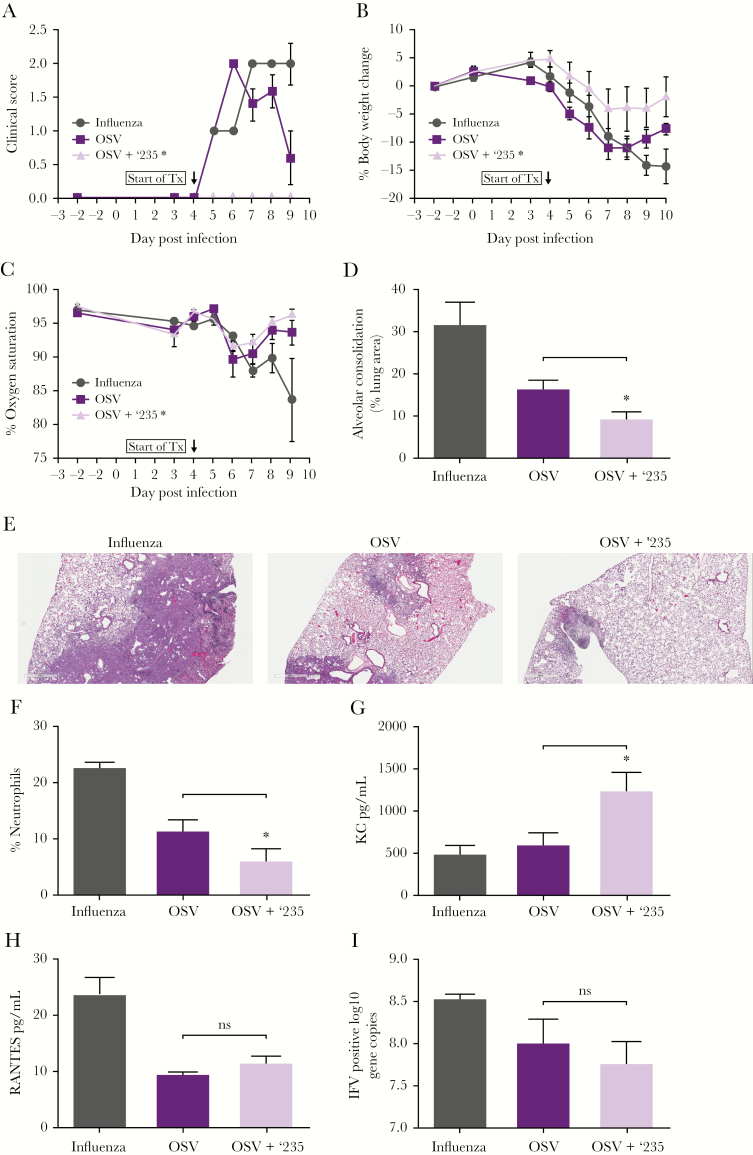
Improvement in clinical symptoms and lung function: mice were infected with H1N1 influenza (A/PR/8/34), and starting on Day 4 postinfection they were treated with placebo, oseltamivir phosphate (OSV), or the combination of the CXCR2 antagonist SB-332235Z (‘235) plus OSV. Mice were evaluated starting 2 days before infection for (A) clinical scores on a 0–3 severity scale (based on appearance, grooming, posture, and activity) through Day 9 postinfection (p.i.) (*average clinical score OSV vs OSV^+^ ‘235, *P* = .0012); (B) body weight changes through Day 10 p.i. (*area under the curve [AUC] body weight change OSV vs OSV^+^ ‘235 *P* = .0176); and (C) oxygen saturation through Day 9 p.i. Improvement in lung consolidation: on Day 14 p.i., lungs were evaluated for (D) histopathologic changes by quantifying areas of alveolar consolidation (*, *P* = .0225) and (E) a representative pictures of hematoxylin and eosin stain (H&E)-stained lungs are depicted. Pathway engagement: bronchoalveolar lavage fluid (BALF) was collected on Day 7. (F) Neutrophils were evaluated by flow cytometry (*, *P* = .028) and protein concentrations of chemokines (G) KC (*, *P* = .0067) and (H) RANTES were measured. Viral load: (I) viral ribonucleic acid levels in the lungs of mice treated with the combination of OSV + ‘235 were similar to the OSV-treated group on Day 7 postinfection. Error bars represent standard error of the mean; 5–6 animals were used per group.

Treatment with the combination of OSV + SB-332235Z led to an overall improvement in the health of the mice compared with OSV monotherapy and placebo. Animals treated with the combination of OSV + SB-332235Z showed significantly improved clinical scores (Figure 1A) and body weight changes (Figure 1B) and a trend towards improved oxygen saturation (Figure 1C) when compared with the OSV monotherapy group.

In addition to clinical measurement of disease improvement, we investigated the impact of treatment on the lungs. Lung pathology analysis on Day 14 postinfection identified distinct areas with histopathologic changes. The affected areas of lung predominately displayed alveolar consolidation, characterized by air spaces and interstitial septae filled or distended by a mixed population of inflammatory cells, necrotic debris, and Type II alveolar epithelial hyperplasia. These changes were consistent with a reparative process potentially leading to restoration of normal alveolar parenchyma. Combination therapy-treated animals had a significant improvement in lung pathology, demonstrated by reduced alveolar injury and consolidation on Day 14 compared with those receiving OSV monotherapy and placebo (Figures 1D and E).

To confirm pathway engagement, groups of mice were euthanized on Day 7 postinfection, after 3 days of treatment. Bronchoalveolar lavage fluid was obtained from the animals and neutrophils were analyzed by flow cytometry. The mice receiving combination treatment had significantly fewer neutrophils in the BALF compared with the placebo and OSV-treated mice, consistent with inhibition of neutrophil chemotaxis towards the site of infection by SB-332235Z (Figure 1F). A significant increase in concentrations of the chemokine KC (CXCR2 ligand and murine homologue of IL-8) in BALF was seen in the combination versus OSV treatment groups, supporting target engagement (Figure 1G). Elevation of KC in murine studies and IL-8 in humans upon CXCR2 antagonism has been shown to be indicative of target engagement, including within the clinical trial that this work helped enable [12]. It is postulated that the reduction of neutrophils at the site of inflammation in the lungs via CXCR2 antagonism diminishes the negative feedback loop and thereby increased production of KC/IL-8, and, in addition, KC/IL-8 is unable to bind to CXCR2 and thereby leads to an increase in detectable KC/IL-8 [13, 14]. Evaluation of the chemokine RANTES, which is unrelated to the CXCR2/KC pathway, revealed no differences between the OSV and combination groups, further confirming target engagement specificity (Figure 1H). Viral RNA levels in the lungs of mice treated with the combination of OSV + SB-332235Z was not significantly changed relative to the OSV-treated group (Figure 1I), indicating that the attenuation of neutrophil recruitment into the lungs did not impair clearance of the virus.

## CONCLUSIONS

We demonstrated that attenuation of neutrophil recruitment to the lungs by therapeutic treatment with the CXCR2 antagonist SB-332235Z together with OSV improved outcomes in a model of severe influenza infection, with no evidence of worsening outcome. These data suggest that attenuation of neutrophil infiltration into the lungs of influenza-infected patients in combination with a DAA would be beneficial over DAA treatment alone. Although OSV was used as the DAA in this study, it is expected that this complementary mechanism of CXCR2 antagonism to reduce lung inflammation would combine well with other agents that reduce viral load. Mechanistically, it is expected that the combination treatment would have the greatest benefit in patients hospitalized with complicated influenza, where neutrophilic inflammation appears to drive disease severity. This population currently has no approved treatment options and therefore represents an area of great unmet medical need. This study helped enable clinical studies to evaluate the effect of CXCR2 antagonism in subjects with influenza [12, 15].
